# Cardiac Catheterization in American Indian/Alaska Native Populations: Empowering Communities, Providers, and Systems Toward Equity

**DOI:** 10.1016/j.jscai.2025.103876

**Published:** 2025-11-11

**Authors:** Joseph Burns, Daniel J. Penny, Keila N. Lopez, Lindsay F. Eilers, Athar M. Qureshi, Christine J. Chung, Jason F. Deen

**Affiliations:** a Section of Cardiology, Department of Pediatrics, Baylor College of Medicine, Texas Children’s Hospital, Houston, Texas; b Division of Cardiology, Department of Medicine, University of Washington, Seattle, Washington; c Division of Cardiology, Departments of Pediatrics and Medicine, University of Washington, Seattle Children’s Hospital, Seattle, Washington

**Keywords:** American Indian/Alaska Native, health inequity, interventional cardiology

## Abstract

American Indian/Alaska Native (AI/AN) people face numerous health disparities, including alarming rates of cardiovascular morbidity and mortality. These inequities are closely tied to unique social, political, and economic circumstances due to the ongoing consequences of systemic racism. Although poorly described, these cardiovascular inequities extend to adult and pediatric interventional cardiology. AI/AN adults are less likely to undergo cardiac catheterization and are more likely to experience complications. There are no published studies on pediatric interventional cardiology outcomes in AI/AN children. The scarcity of available data underscores the need for novel studies to investigate the epidemiology, sociodemographic associations, and outcomes of AI/AN adults and children who require cardiac catheterization. Additionally, interventions addressing the geographic and social barriers to care require thoughtfulness and cultural sensitivity. Equity in interventional cardiology for AI/AN people may be best achieved through partnerships among tribes, health care providers, industry, health systems, and regulatory bodies, as well as accurate data collection. A concerted effort is required to meet the needs of this vulnerable population.

## American Indian/Alaska Native health inequity and social, economic, and political contexts

American Indian/Alaska Native (AI/AN) people comprise nearly 3% of the population of the United States and are disparately affected by numerous health inequities, including substance use, diabetes, cancer, injuries, and cardiovascular disease (CVD).^1^^,^^2^ CVD morbidity and mortality in AI/AN populations alarmingly represent the leading cause of death (19.2%).^3^^,^^4^ Risk factors for CVD disproportionately impact AI/AN youth, and the longitudinal consequences of this disparate exposure manifest as early-onset CVD.^3^

Poorer CVD outcomes in AI/AN populations are closely related to the ongoing legacy of settler colonialism in the United States and downstream structural racism. AI/AN populations have been forcibly relocated with consequent loss of life, culture, and language.^5^ Loss of traditional practices and diet has promoted increased reliance on high-fat, carbohydrate-laden commodity foods.^6^ Structural racism continues to affect health outcomes in the current era through land allocation patterns and unequal distribution of federal funding, all of which have downstream impacts, including access and payment for health care, ongoing threats to the environment and tribal lands, and violence, including the Missing and Murdered Indigenous People crisis.^3^^,^^5^ Further, social, economic, and political contexts are particularly relevant, as the ongoing consequences of structural racism manifest as targeted stressors for AI/AN populations. These stressors promote the physiologic and psychological mechanisms that contribute to cardiometabolic disease.^7^ This article aimed to summarize CVD inequities in AI/AN adults and children, review existing literature on cardiac catheterization in these populations, and offer strategies to improve cardiovascular health, access to catheterization, and outcomes for this population.

## AI/AN adult CVD inequity

The significant burden of CVD in AI/AN people has been well reported and summarized in a 2020 Scientific Statement from the American Heart Association.^4^ Since the 1980s, studies have described inequitable CVD incidence in AI/AN adults.^8^ From 1987-1996, higher incidences of coronary artery disease (106.65 per 100,000 to 511.07 per 100,000) and myocardial infarction (35.55 per 100,000 population to 283.93 per 100,000) were found in the White Mountain Apache Tribe of Arizona.^9^ More alarmingly, this study also found that 49% of a control sample with a mean age of 27.3 years had 2 or more CVD risk factors.^9^

The Strong Heart Study (SHS), a large, prospective, longitudinal cohort study in AI people, provides the most robust literature on CVD in AI/AN people. The SHS included over 3500 AI persons in 13 tribes and described that high-density lipoprotein decreased, systolic blood pressure increased, and diabetes increased over a follow-up of 2-4 years.^10^ In addition, coronary artery disease morbidity and mortality in the SHS study exceeded rates of coronary artery disease in other demographics.^11^ Fortunately, in a 25-year follow-up, declines were noted in both the incidence of and mortality due to CVD, reflecting the strength and efficacy of community-based programming, including improved access to high-quality care, adherence to medical regimens, and increased attention paid to the social drivers of CVD.^12^

Additionally, exposure to air pollution, water pollution, and heavy metals has demonstrated an association with the development of CVD.^8^ SHS investigations revealed higher serum and urine concentrations of multiple heavy metals, including cadmium and arsenic.^8^ These exposures are further complicated by infrastructural limitations to access to electricity and clean water.^5^^,^^8^

## AI/AN pediatric cardiovascular inequity

A growing body of literature describes inequities in congenital heart disease (CHD) and early-onset CVD in non-White populations. Black children demonstrate higher odds of mortality after congenital heart surgery, and patients from lower socioeconomic status are less likely to be diagnosed prenatally.^13^ Further, geographical distance to a specialized cardiac center has also been associated with poorer outcomes, with a higher incidence of mortality for infants of mothers who are more remote from centers.^14^ Recent scientific articles and calls to action fail to specifically report CVD and congenital cardiac epidemiology and outcomes in AI/AN children.

The Strong Heart Family Study (SHFS), an expansion of the SHS cohort in its fifth phase, includes a large sample of adolescent patients who are family members of adult SHS participants.^8^ In this study, 24.9% of adolescents met the criteria for metabolic syndrome.^8^ The AI/AN youth with CVD risk factors demonstrated subclinical CVD, including left ventricular hypertrophy or dysfunction.^8^ A recent SHFS evaluation found that body mass index increased while ejection fraction decreased among overweight/obese AI men, demonstrating 3.6 times higher odds of impaired ejection fraction than those with normal body mass index.^15^

Another SHFS evaluation found dyslipidemia in 55.2% of participants between the ages of 15 and 18 years.^16^ Even in this young population, there was an unexpectedly high rate of CVD events,^16^ with contributing factors including poor diet due to reliance on commodity foods, increasing incidence of diabetes mellitus and obesity, tobacco use, and limited physical activity.^6^^,^^8^^,^^17^

## AI/AN inequity in cardiac catheterization

### Access and outcomes in AI/AN adult interventional cardiology

Recent studies have sought to describe inequities in cardiac catheterization access and outcomes, particularly those specific to AI/AN patients, although these are limited. A study evaluating outcomes in percutaneous coronary interventions reported a higher all-cause cardiac complication rate in AI/AN men and the highest rate of stent thrombosis for AI/AN women.^18^ Importantly, evidence suggests that there is no significant increase in prevalence of CYP2C9 variant alleles, which limit the capacity to activate clopidogrel, in AI populations, to limit the efficacy of this medication.^19^ However, small studies suggest that among AI patients on low-dose aspirin, ticagrelor demonstrated faster onset and greater platelet suppression than clopidogrel.^20^ This inequity may also be compounded by limited medication access and adherence, as AI/AN populations have demonstrated lower adherence to antihypertensive medications than other groups, particularly in rural settings.^21^ Further, although the all-cause mortality increased in all demographics, the AI/AN women demonstrated a statistically significantly higher increase than White populations.^18^

An evaluation using the Nationwide Inpatient Sample and the Indian Health Service National Patient Information Reporting System compared rates of surgical and interventional procedures following acute myocardial infarction in AI/AN patients relative to White populations.^22^ When stratified by geographic regions, this study found that AI/AN patients in 3 of 4 geographic regions were less likely than White patients to undergo catheterization.^22^ Among diabetic patients, AI/AN individuals were less likely to undergo coronary artery bypass grafting.^22^

On the contrary, in a cross-sectional study using California patient discharge data, including 796 hospitalizations for AI/AN adults with ischemic heart disease, AI/AN patients did not have lower rates of catheterization or percutaneous intervention relative to the White sample.^23^

### Pediatric interventional cardiology in AI/AN populations

Data describing pediatric interventional cardiac care for AI/AN children are absent. No interventional catheterization study has focused on this population, nor, to the best of our knowledge, have there been any independent reports on rates of AI/AN cardiac catheterization. In aggregated data, AI/AN patients are often grouped with other populations due to sample size limitations, which hinders the extrapolation of results specific to this population. Recent summaries and calls to action also fail to explicitly address potential inequities for AI/AN children in pediatric cardiac catheterization.^24^^,^^25^

There is recent evidence that suggests there is a higher incidence of critical CHD in AI/AN infants.^26^ Further, there has been a growing trend of treatment of congenital heart defects via interventional cardiology techniques versus surgery in recent years, including in the neonatal population with critical CHD.^27^ Given these findings, studying the access to cardiac catheterization and relevant outcomes in the AI/AN population with CHD is of utmost importance.

## Strategies to improve AI/AN cardiac catheterization access and equity in procedures in adults and children

Rurality presents significant limitations to accessing catheterization services, which is particularly impactful among AI/AN populations. There are approximately 125 pediatric cardiology programs in the United States, with 8 states without a center.^28^ The implementation of mobile cardiac catheterization laboratories has improved access to care since 1987.^29^ Evaluations of this model in North Carolina demonstrated increased access for women and Black patients but found that AI patients were more frequently served as outpatients at Duke University Medical Center rather than at a mobile laboratory (2.3% vs 1.2% of cases).^29^

Mobilizing providers to communities may offer a potential strategy for improving access to care in AI/AN communities. Prioritizing partnerships with local and tribal communities, as well as governments, and including community members in these efforts is crucial to promoting sustainability and a community-sensitive and culturally sensitive approach. Generally, such efforts addressing not only catheterization but also diagnostic echocardiography and clinical cardiology evaluation may improve the significant inequities facing AI/AN people. In children, a mobile model of care presents substantial challenges due to limited patient volume, the unique supply and anesthetic considerations, and the necessity of surgical backup for complex cases. However, a mobile laboratory may be feasible for lower-risk diagnostic procedures and interventions, particularly if well-planned and coordinated with effective partnerships between local communities, providers, and the supporting medical center.

However, rurality is not a limitation for AI/AN individuals residing in urban settings. Approximately 71% of the AI/AN population resides in urban areas.^4^ Importantly, heart disease is the leading cause of mortality for the urban AI/AN population and is higher than that for the urban White population.^30^ Alarmingly, a survey of urban AI/AN adults found that respondents recognized approximately half of the queried symptoms of myocardial infarction and stroke, far less than the general population.^31^ Urban AI/AN individuals are uniquely vulnerable, as funding for urban AI/AN health represents only 1% of the Indian Health Service allocation, and individuals may be disconnected from reservation-based health services.^32^ It is therefore critical to recognize that impaired access to health services presents limitations to interventional cardiology care even among urban AI/AN populations.

Evaluations specific to CHD in AI/AN people are lacking. Unfortunately, AI/AN patients are often not reported independently and may be aggregated with multiple populations as “other.”^33^ This misrepresentation fails to provide a true understanding of AI/AN epidemiology and outcomes and limits the pooling of data from multiple centers in more extensive evaluations or meta-analysis.^33^

In addition, assessing the representation of AI/AN people in the cardiovascular workforce is critical, as racial concordance between patients and providers is associated with improved outcomes.^13^ In the field of pediatric cardiology, in 2023, no physicians indicated AI/AN as their only racial/ethnic identity, and 15 reported 1 or more groups.^34^ This is a stark underrepresentation, considering 3.7% of the children in the United States are AI/AN.^35^ This is compounded by a lack of AI/AN faculty mentors in academic medicine.^36^ For this reason, the active recruitment of AI/AN trainees and the retention of AI/AN faculty may promote representation in adult and pediatric cardiology, benefiting the AI/AN patients served.^36^

Community engagement through continued communication and collaboration among stakeholders in interventional cardiology (including providers, industry, health systems, and regulatory bodies), as well as tribal leaders, must also consider incorporating tribal perspectives to better understand and mitigate these inequities. Such conversations have proven effective in addressing the current landscape and needs in the area of pediatric valvular disease and may also provide an effective forum for patient and community perspectives in both pediatric and adult interventional cardiology.^37^ These approaches are summarized in the Central Illustration.

**Central Illustration:**
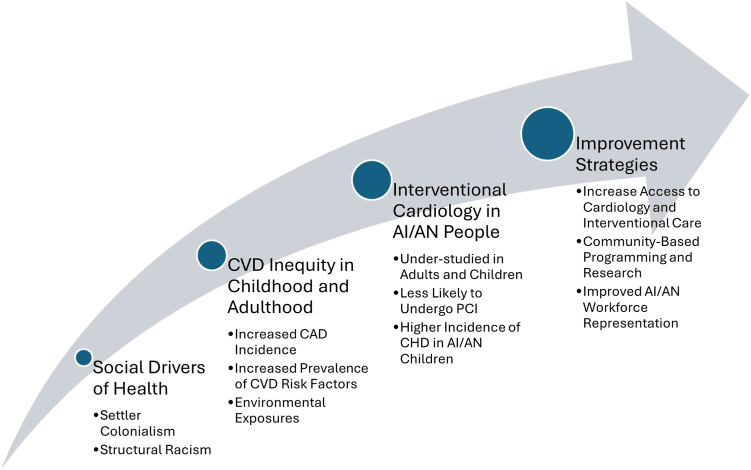
**Visual representation of social contexts and inequity in AI/AN CVD and cardiac catheterization.** AI, American Indian; AN, Alaska Native; CHD, congenital heart disease; CVD, cardiovascular disease.

## Conclusion

Cardiac catheterization studies reporting AI/AN-specific data are lacking. It is essential to support such investigations of this specific population to better understand their unique needs and potential inequities. Ultimately, all providers and health systems must promote health equity. At a population level, this includes partnerships with tribes and communities to understand priorities, historical experience, and active efforts to address cultural preservation and health promotion.^17^ Additionally, cardiology-specific studies and assessments in AI/AN populations, specifically designed to identify and address risk factors for CVD, are crucial for further understanding the needs of this population. Understanding the health care systems with which AI/AN patients and families interface is paramount to improving access to high-quality, timely interventional cardiac care.
